# *Clonorchis sinensis* infection remodels chromatin accessibility in hepatocellular carcinoma

**DOI:** 10.1186/s13071-025-06909-6

**Published:** 2025-07-10

**Authors:** Weilong Yang, Caibiao Wei, Junxian Chen, Qiumei Lin, Yuling Qin, Taijun Huang, Xueling Deng, Mulin Jun Li, Zeli Tang, Min Fang

**Affiliations:** 1 Department of Clinical Laboratory, Guangxi Medical University Cancer Hospital, Nanning, 530021 Guangxi China; 2 Guangzhou Women and Children’s Medical Center, Guangzhou Medical University, Guangzhou, China; 3 Department of Cell Biology and Genetics, School of Basic Medical Sciences, Guangxi Medical University, Nanning, 530021 China; 4 Key Laboratory of Basic Research on Regional Diseases (Guangxi Medical University), Education Department of Guangxi Zhuang Autonomous Region, Nanning, 530021 China; 5 Guangxi Clinical Research Center for Anesthesiology, Guangxi Medical University Cancer Hospital, Nanning, 530021 Guangxi China

**Keywords:** *Clonorchis sinensis*, Hepatocellular carcinoma, Chromatin accessibility, Transcription factor, Tumor progression

## Abstract

**Background:**

Hepatocellular carcinoma (HCC) is a major global health concern, accounting for a significant proportion of liver cancer cases and related deaths. *Clonorchis sinensis* (*C. sinensis*) infection, a recognized carcinogen, has been implicated in the progression of liver diseases, including HCC. However, the precise epigenetic mechanisms underlying *C. sinensis*-associated HCC remain to be elucidated.

**Methods:**

To investigate the role of chromatin accessibility in *C. sinensis*-related HCC progression, we performed an assay for transposase-accessible chromatin with high-throughput sequencing (ATAC-seq) and RNA sequencing (RNA-seq) analyses of *C. sinensis*-infected (*C. sinensis*^+^) and non-*C. sinensis*-infected (*C. sinensis*^−^) HCC tumors. Integrated analyses were conducted to assess chromatin accessibility, transcription factor (TF) motifs, and histone modifications using ATAC-seq, RNA-seq, and classical chromatin immunoprecipitation-sequencing (ChIP-seq) datasets. A scratch wound assay was used to evaluate the effects of *C. sinensis* excretory/secretory products (*Cs*ESPs) on HCC cell migration.

**Results:**

ATAC-seq analysis revealed 9,396 differentially accessible regions (DARs) in *C. sinensis*^+^ HCC tumors compared with *C. sinensis*^−^ HCC tumors. Additionally, several crucial TFs enriched in DARs were identified, including HNF4A, FOXO1, ELF4, and RELA. Combined ATAC-seq and RNA-seq analyses further revealed differentially expressed genes (DEGs) associated with metabolism, immune regulation, and cytoskeletal dynamics. Chromatin accessibility was closely associated with histone modifications such as H3K9ac, H3K4me2, H3K4me3, H3K27ac, H3K4me1, and CTCF binding. Notably, *C. sinensis* infection significantly increased the migratory capacity of HCC cells, as confirmed by molecular assays and clinical observations.

**Conclusions:**

Our study demonstrates that *C. sinensis* infection remodels chromatin accessibility and may contribute to HCC progression. Our work offers valuable insights into the pathogenesis of HCC in the context of parasitic infection and lays the groundwork for future biomarker and therapeutic target discovery.

**Graphical abstract:**

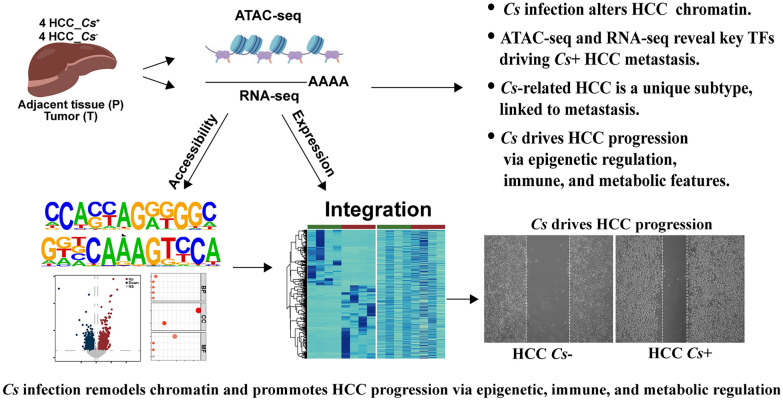

**Supplementary Information:**

The online version contains supplementary material available at 10.1186/s13071-025-06909-6.

## Background

Liver cancer was the sixth most common cancer and the third leading cause of cancer-related deaths worldwide in 2022, with approximately 870,000 new cases and 760,000 deaths annually [1]. Hepatocellular carcinoma (HCC) is the most common type of primary liver cancer, accounting for 70–85% of cases [2]. Major risk factors for HCC include viral hepatitis and cirrhosis, metabolic liver disease (e.g., diabetes mellitus and obesity), and persistent intake of foods containing dietary toxins such as aflatoxin and aristolochic acid, which significantly increase the risk of HCC by either directly damaging the liver or triggering associated inflammation [3]. Although current treatments have significantly improved the survival rates of patients with HCC, the prognosis for patients with advanced stages of the disease remains poor.

Significantly, substantial data suggest that the high prevalence of helminth infections worldwide significantly increases the susceptibility of numerous populations to the risk of cancer [4], and *Clonorchis sinensis* (*C. sinensis*) infection has been classified as a definite group I carcinogen by the International Agency for Research on Cancer (IARC) [5]. Most cases of clonorchiasis arise when humans or other mammals ingest raw or undercooked freshwater fish and shrimp containing metacercariae; the mature worm lives primarily in the intrahepatic bile duct, common bile duct, or gall bladder [6]. Infection with *C. sinensis* can provoke severe tissue damage and pathological changes, such as obstructive jaundice, cholangitis, cholestasis, cholelithiasis, and even cholangiocarcinoma (CCA) [7]. Unfortunately, an increasing number of studies have confirmed the important role of *C. sinensis* in the progression of HCC, in both humans and mice [8–11]. Furthermore, *C. sinensis* granulin (*Cs*GRN), a growth factor-like protein from *C. sinensis* excretory/secretory products (*Cs*ESPs), has been reported to affect host cell functions and immune responses and promote the malignant transformation of hepatocytes [12]. Although accumulated evidence suggests that *C. sinensis* can promote the development of HCC through various pathways, the specific mechanisms underlying these alterations, particularly the dynamics and regulatory roles of chromatin accessibility, which have not yet been reported, are still unclear.

Chromatin, through dynamic regulation by transcription factors (TFs), plays a central role in influencing cell fate and transcriptional programs [13]. Under normal physiological conditions, key TFs control lineage determination pathways and regulate cell fate by modulating gene expression through chromatin accessibility, thus establishing distinct cellular phenotypes [14]. In cancer, the concept of transcriptional addiction highlights that cancer cells exhibit heightened sensitivity to the impairment of specific TFs, underscoring their complex role in regulating cellular outcomes [15]. Many studies have emphasized the importance of epigenetics in the pathogenesis of HCC [16–18]; however, given the multifactorial nature of the pathogenesis of HCC, the functional impacts of *C. sinensis*-associated epigenetic dysregulation and their interactions with genomic alterations, especially in chromatin accessibility, remain unclear in HCC. Consequently, we first conducted comprehensive profiling of *C. sinensis-*infected (*C. sinensis*^+^) and non-*C. sinensis*-infected (*C. sinensis*^−^) HCC cells by integrating an assay for transposase-accessible chromatin with high-throughput sequencing (ATAC-seq), RNA sequencing (RNA-seq), and classical chromatin immunoprecipitation-sequencing (ChIP-seq) data to systematically investigate the changes in regulatory epigenomes and their associations with *C. sinensis* infection and genomic alterations in HCC patients.

## Methods

### Human samples

Liver tissue samples were obtained from treatment-naive patients with HCC who underwent surgical resection at the Department of Hepatobiliary Surgery, Affiliated Cancer Hospital of Guangxi Medical University (Nanning, China), where all patients had not received any treatment prior to surgery.

In our study, tumor tissues were obtained from nodular lesions with typical macroscopic features observed during surgical resection. Each tumor sample was histopathologically confirmed by experienced pathologists to ensure the presence of malignant HCC cells. Paired tumor-adjacent tissues were collected from areas at least 5 cm away from the visible tumor margin that showed no macroscopic signs of tumor infiltration. These adjacent tissues were also subjected to histopathological examination to confirm the absence of malignant or dysplastic cells, ensuring a clear distinction between the tumor and nontumor regions.

### Study population and data collection

A total of 2390 patients diagnosed with HCC underwent curative resection between July 2013 and December 2022. The inclusion criteria for this retrospective study were as follows: patients with (1) HCC confirmed by postoperative pathology who underwent surgical resection, (2) no prior anticancer treatments, (3) no presence of additional malignant tumors, and (4) complete laboratory and pathological data available. The exclusion criteria were as follows: (1) 267 patients who had received antitumor therapy (e.g., radiation or chemotherapy), (2) 115 cases without a confirmed pathologic diagnosis, (3) 229 patients with other tumor-related diseases, (4) 201 patients with recurrent HCC, and (5) 631 cases who because of incomplete laboratory, follow-up, transfer data or in the perioperative period.

The diagnostic criteria for clonorchiasis included any of the following conditions: (1) intraoperative or postoperative pathological examination detecting adult *C. sinensis* in the liver or gallbladder, and (2) preoperative fecal examination revealing *C. sinensis* eggs. Metastatic surveillance in postoperative patients included regular imaging computed tomography (CT), magnetic resonance imaging (MRI), or positron emission tomography (PET) and clinical evaluation to monitor for the development of metastases, which may appear in extrahepatic organs (e.g., lungs, bones, and lymph nodes) or in different parts of the liver. In addition, confirmation of metastatic lesions was performed by tissue biopsy, including intraoperative pathology or postoperative pathological analysis of resected samples, to observe the presence of primary tumor cells in other tissues.

### Collection and preparation of *Cs*ESPs

To collect *Cs*ESPs, *C. sinensis* metacercariae were harvested from *Pseudorasbora parva* (naturally infected freshwater fish) in Hengxian County, Guangxi, China. The fish were processed by removing nonmuscle tissues, deboned, minced, and digested overnight at 37 °C in 0.8% pepsin with 0.2% HCl. The mixture was filtered through a 60–80 mesh sieve, and live metacercariae were isolated microscopically and stored in phosphate-buffered saline (PBS) at 4 °C. Adult *C. sinensis* worms were collected from the bile ducts of infected Sprague–Dawley (SD) rats. The worms were cultured with medium that was changed every 6 or 12 h. After 48 h of culture, the medium was pooled. The mixture was subsequently centrifuged at 12,000 rpm for 30 min at 4 °C, dialyzed in PBS, and concentrated using sucrose or lyophilization based on intended use. The weights and concentrations of the samples were measured, and the samples were stored at −80 °C. Before use, the samples were filtered through a 0.22-µm ultrafiltration membrane to ensure sterility.

### Scratch wound experiment

The HCC cell line HCCLM3 was obtained from the Type Culture Collection of the Chinese Academy of Sciences (Shanghai, China). The cells were seeded in six-well plates at a density of 5 × 10^5^ cells per well and incubated for 24 h in Dulbecco’s Modified Eagle Medium (DMEM) supplemented with 10% heat-inactivated fetal bovine serum (Wisent, Canada) in a humidified incubator at 37 °C with 5% CO_2_. Once the cells reached 80–90% confluence, the culture medium was removed, and the floating cells were washed twice with 1 × PBS (Gibco, USA) to remove any residual medium. To create a scratch wound, a 10 µL pipette tip was used to make three horizontal scratches in each well. After the cells were washed with 1 × PBS to remove floating debris, they were cultured in complete medium supplemented with different substances. In the experimental group, *Cs*ESPs were added at a concentration of 50 μg/mL, while the control group received an equal volume of 1 × PBS. Imaging was performed using a light microscope (ZEISS Axio Vert.A1) immediately after adding the treatment media (0 h), followed by further observations at 24 h intervals for a total of 72 h. Scratch areas were imaged at low magnification to record and analyze the migration of the HCCLM3 cells over time.

### ATAC-seq and RNA-seq

A total of 50,000 nuclei were added to 1 mL of washing buffer and centrifuged at 500 × *g* for 5 min at 4 °C. Tn5 transposase was added, and the transposition reaction was incubated at 37 °C for 30 min. Equimolar adapters 1 and 2 were added after transposition, and Polymerase Chain Reaction (PCR) was then performed to amplify the library. After PCR, the libraries were purified with AMPure beads, and library quality was assessed with a Qubit instrument. Clustering of the index-coded samples was performed on a cBot Cluster Generation System using the TruSeq PE Cluster Kit v3-cBot-HS (Illumina, China) according to the manufacturer’s instructions. The library preparations were sequenced on the Illumina NovaSeq platform at Tianjin Novogene Bioinformatic Technology Co., Ltd. (Beijing, China), and 150-bp paired-end reads were generated.

RNA sequencing library preparation was performed following the protocols provided by the manufacturer. Total RNA was extracted using an RNA extraction kit. Briefly, rRNA depletion was employed to enrich mRNAs and noncoding RNAs from total RNA. Both mRNAs and noncoding RNAs were subsequently fragmented into short segments (~200–700 bp). The initial Complementary DNA (cDNA) strand was synthesized using a random hexamer primer, followed by the synthesis of the second cDNA strand with DNA polymerase I. After purification, the cDNA fragments were ligated with sequencing adapters. The second cDNA strand underwent degradation facilitated by uracil-N-glycosylase (UNG). The final cDNA library was generated by PCR after DNA isolation and purification and then quantified by an Agilent 2100 instrument. The cDNA library was sequenced using a HiSeq Xten device according to commercial protocols.

### Analysis of ATAC-seq data

Trim Galore (v.0.6.10) [19] was used to remove low-quality reads and adapters from the raw sequencing reads and obtain clean data. Clean data were aligned to the hg38 reference genome using Bowtie2 (v.2.5.1) [20] with the following options: --very-sensitive -X 2000. The PCR duplicates were removed by sambamba (v.0.6.6) [21]. Moreover, mitochondrial DNA was also deleted. DeepTools bamCoverage was used to transform alignment bam files into bigwig files with the following options: --normalizedUsing RPKM. MACS2 was used to call peaks with the following parameters: -g hs --nomodel --shift -100 --extsize 200. The Integrative Genomics Viewer (IGV) (v.2.16.1) [22] was used for representative region visualization. Differentially accessible peaks were identified by using the R package DEseq2 (v.1.44.0) (|fold change|> 2 and *P* < 0.05) [23]. Motif enrichment analysis was performed with the HOMER function findMotifsGenome.pl with the default option (*P* < 0.01). The function annotatePeak of the R package ChIPseeker (v.1.34.1) [24] was used to annotate the peaks. Gene Ontology (GO) and Kyoto Encyclopedia of Genes and Genomes (KEGG) enrichment analyses were performed using the R package clusterProfiler (v.4.12.0) [25].

### Analysis of RNA-seq data

Clean data were obtained by removing low-quality reads and adapters from raw sequencing reads with Trim Galore (v.0.6.10) [19]. HISAT2 was used to align the clean data to the hg38 reference genome with default parameters. The gene expression matrix was built with featureCounts (v.2.0.6) [26]. The R package DEseq2 (v.1.44.0) [23] was employed to normalize gene expression and detect differentially expressed genes (|fold change|> 2 and *P* < 0.05). GO and KEGG enrichment analyses were performed using the R package clusterProfiler (v.4.12.0) [25].

### Integrative analysis of RNA‑seq and ATAC‑seq data

The function annotatePeak of the R package ChIPseeker (v.1.34.1) [24] was used to annotate the differentially accessible peaks to the nearest transcription start sites (TSS) to evaluate the association between chromatin accessibility and gene expression changes. Regions within 3 kb upstream and downstream of the TSS were considered promoter regions. Finally, the number of reads in all peaks related to promoter regions was computed for each gene as the gene activity score. The parameters of the DEGs and gene activity scores were |log2(fold change)|> 0.5 and *P* < 0.05.

### Integrative analysis of ChIP‑seq and ATAC‑seq data

The bigwig files from ChIP-seq in the HepG2 cell line were downloaded from the GEO database (GSE29611). CrossMap (v.0.7.0) [27] was used to perform genome coordinate conversion from hg19 to hg38. Deeptools computeMatrix was used to analyze enrichment signals of ChIP-seq and ATAC-seq, and deeptools plotHeatmap was used to present enrichment signals. The IGV (v.2.16.1) [22] was used for representative region visualization.

### Statistical analysis

R software (v.4.3.0) was used for statistical analysis. The correlation between variables was evaluated by Pearson’s test, and statistical significance was set at *P* < 0.05. Statistical tests were conducted with GraphPad Prism version 8.0.2. Data are presented as mean ± standard deviation of three independent experiments. Intergroup differences for categorical data presented as ratios were compared using the Chi-squared test. Independent samples were analyzed using a *t*-test or Analysis of Variance (ANOVA) to assess differences between groups. A *P* value of < 0.05 was considered to indicate statistical significance; *P < 0.05.

## Results

### Quality of ATAC-seq data from *C. sinensis*^+^ and *C. sinensis*^−^ HCC

**Fig. 1:**
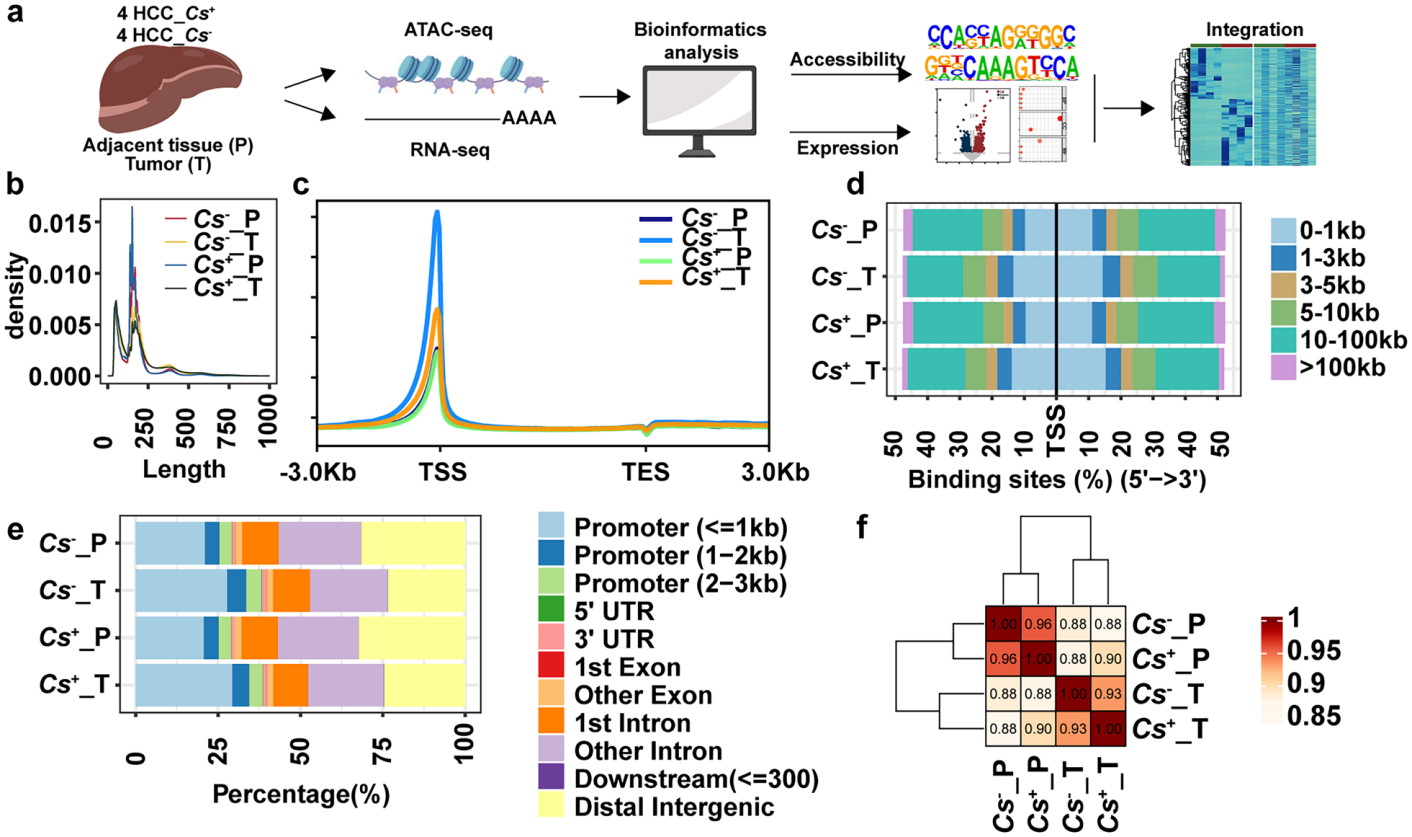
Chromatin accessibility landscape of *C. sinensis*^+^ and *C. sinensis*^−^ HCC. **a**. Experimental strategy for genome-wide ATAC-seq assay and integration analysis of *C. sinensis*^+^ and *C. sinensis*^−^ HCC. **b**. Fragment size of ATAC data in each group of *C. sinensis*^+^ and *C. sinensis*^−^ HCC. **c**. Distribution of peaks relative to TSS and TES regions in each group. **d**. Distribution of reads from ATAC-seq data relative to TSS in each group. **e**. Genomic distribution of reads from ATAC-seq data in each group. **f**. Correlation analysis between all groups. *Cs* represents* C. sinensis*

To define the influence of *C. sinensis* infection on the chromatin accessibility of HCC, a workflow was designed (Fig. 1a). Four pairs of *C. sinensis*^+^ HCC tumor (*C. sinensis*^+^_T) and adjacent tissue (*C. sinensis*^+^_P) samples and four pairs of *C. sinensis*^− ^HCC tumor (*C. sinensis*^−^_T) and adjacent tissue (*C. sinensis*^−^_P) samples were subjected to ATAC-seq. The information for all patients is listed in Supplementary Table S1. Signals from accessible regions were obtained by alignment, duplication removal, and peak calling. Finally, 168,548 regions were obtained (Supplementary Table S2). The chromatin accessibility fragments exhibited a dual nucleosome peak (Fig. 1b). ATAC-seq signals were enriched mainly in the TSS region, indicating their high quality (Fig. 1c). In addition, the distribution of regions in all groups is also shown (Fig. 1d, e). Quality control analyses confirmed that the ATAC-seq data met the established quality standards. Finally, we also performed correlation analysis, and the results revealed that *C. sinensis*^+^_T and *C. sinensis*^−^_T were more similar and that *C. sinensis*^+^_P and *C. sinensis*^−^_P were more similar (Fig. 1f). All analyses were also performed at the sample level (Supplementary Fig. S1).

### Profile of chromatin accessibility in *C. sinensis*^+^ and *C. sinensis*^−^ HCC

**Fig. 2:**
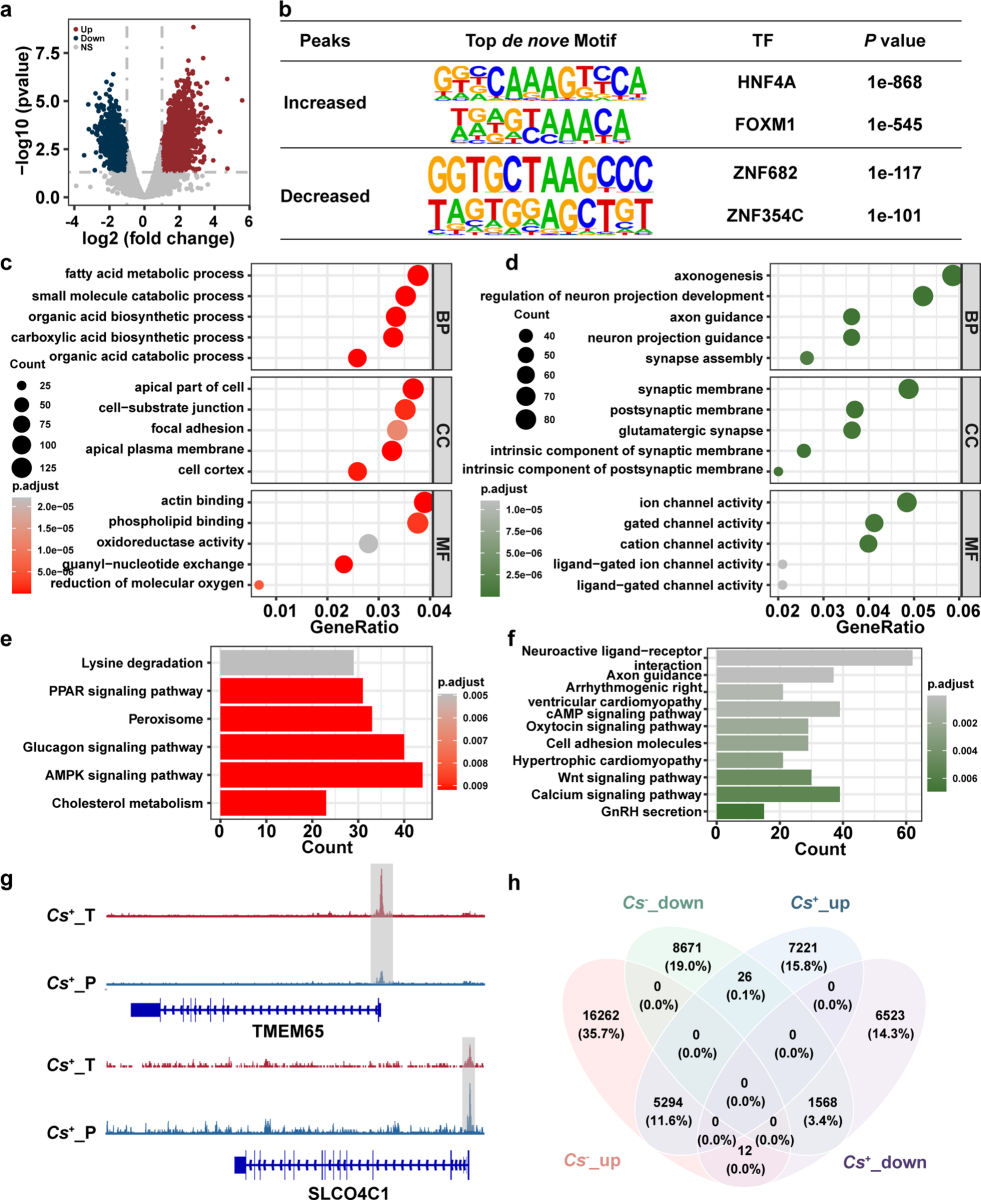
Chromatin accessibility landscape of *C. sinensis*^+^ HCC. **a**. Different chromatin accessibility landscape between *C. sinensis*^+^ HCC tumors and tumor-adjacent tissues. **b**. The top de novo motifs enriched in up and down accessibility peaks between *C. sinensis*^+^ HCC tumors and tumor-adjacent tissues. **c**, **d**. Enrichment analysis of GO terms for up (**c**) and down (**d**) accessibility peaks between *C. sinensis*^+^ HCC tumors and tumor-adjacent tissues. **e**, **f**. KEGG analysis of up (**e**) and down (**f**) accessibility peaks between *C. sinensis*^+^ HCC tumors and tumor-adjacent tissues. **g**. IGV shows representative difference peaks between *C. sinensis*^+^ HCC tumors and tumor-adjacent tissues. **h**. Venn diagram for the relationship between different peaks in *C. sinensis*^+^ and *C. sinensis*^−^ HCC. *Cs* represents* C. sinensis*

We first explored the chromatin accessibility of *C. sinensis*^+^ HCC and identified the different accessibility regions (DARs). The results revealed that 12,541 regions were positively enriched, whereas 8103 regions were negatively enriched in *C. sinensis*^+^ HCC tumors compared with adjacent tissues, of which differentially enriched regions accounted for 12.25% of the overall regions identified (Fig. 2a). Next, motif analysis of regions with increased and decreased accessibility was performed. The top 2 highly expressed TFs (HNF4A and FOXM1) whose motifs were enriched in regions with increased accessibility and the top 2 downregulated TFs (ZNF682 and ZNF354C) whose motifs were enriched in regions with decreased accessibility in *C. sinensis*^+^ HCC tumors are shown in Fig. 2b. GO analysis revealed that the regions with increased chromatin accessibility in *C. sinensis*⁺ HCC tumors were enriched predominantly in biological processes related to fatty acid metabolism, small molecule catabolism, and organic acid biosynthesis. These pathways are closely linked to cellular metabolism, and their enrichment indicates metabolic reprogramming, which is a well-established hallmark of HCC initiation and progression [28]. Cellular component terms such as cell–substrate junction, focal adhesion, and apical plasma membrane features are often associated with epithelial–mesenchymal transition (EMT) and increased invasive potential [29, 30] (Fig. 2c). In contrast, regions with decreased accessibility were significantly enriched for genes involved in axonogenesis and ion channel activity (Fig. 2d). While typically associated with neural function, these pathways have been increasingly implicated in cancer progression [31–34]. For example, ion channels and neurotransmitter pathways can modulate intracellular calcium, cell migration, and tumor–stroma interactions, suggesting the potential functional suppression of these programs in *C. sinensis*⁺ HCC. KEGG pathway analysis corroborated these findings. Regions with increased chromatin accessibility were enriched in the PPAR signaling [35, 36], AMPK signaling and peroxisome pathways [35, 37], all of which are central to lipid metabolism and oxidative homeostasis. These findings further support the presence of a metabolically reprogrammed tumor phenotype (Fig. 2e). In contrast, regions with decreased chromatin accessibility were enriched in neuroactive ligand‒receptor interactions, axon guidance, and cardiomyopathy-associated pathways (Fig. 2f). Finally, two representative regions of altered chromatin accessibility were identified (Fig. 2g).

The DARs between tumors and adjacent tissue in *C. sinensis*^− ^HCC were subsequently analyzed, and 21,568 increased and 10,265 decreased accessibility regions were identified in *C. sinensis*^− ^HCC tumors compared with adjacent tissue, accounting for 18.89% of the overall regions identified (Supplementary Fig. S2a). The top 2 TFs whose motifs were enriched in increased-accessibility regions were CTCF and FOXM1, and the top 2 TFs whose motifs were enriched in decreased-accessibility regions were ZNF682 and ZNF274 (Supplementary Fig. S2b). After GO analysis, increased-accessibility regions were enriched for genes involved in the Wnt signaling pathway, cell-substrate junction, and kinase activity, etc. (Supplementary Fig. S2c). However, regions with decreased accessibility were enriched for genes involved in axonogenesis, positive regulation of cell development, and axon guidance, etc. (Supplementary Fig. S2d). The results of the KEGG analysis revealed that increased-accessibility regions were enriched for genes involved in viral carcinogenesis and the cell cycle, etc. (Supplementary Fig. S2e), and decreased-accessibility regions were enriched for genes involved in the calcium signaling pathway and axon guidance, etc. (Supplementary Fig. S2f). Finally, we compared the regions with increased and decreased accessibility (tumors versus adjacent tissue) in *C. sinensis*^+^ and *C. sinensis*^−^ HCC, and a marked difference was detected, suggesting that *C. sinensis* infection remodels chromatin accessibility in HCC (Fig. 2h).

### Characterization of chromatin dynamics in *C. sinensis*⁺ and *C. sinensis*⁻ HCC tumors

**Fig. 3:**
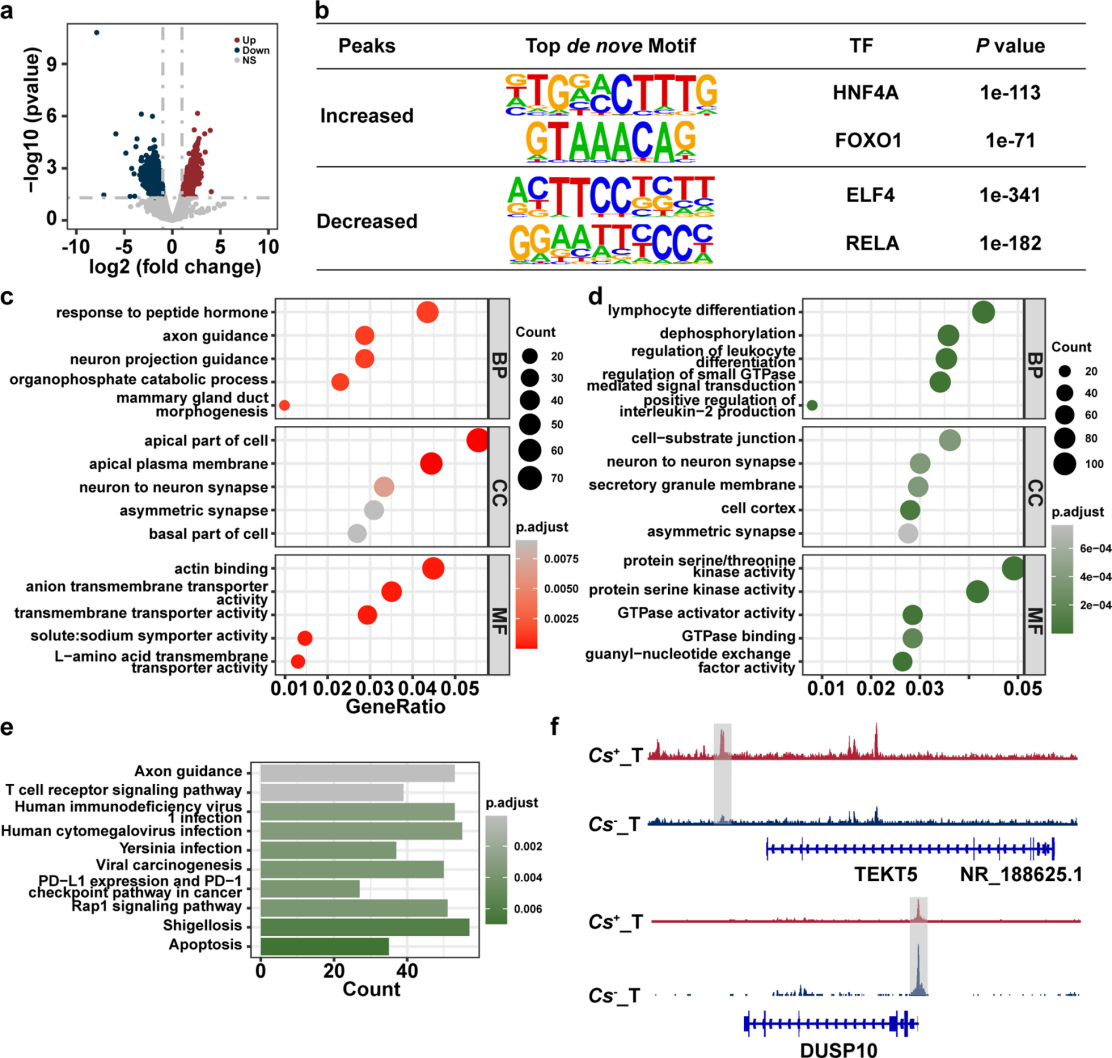
*C. sinensis* infection changes the chromatin accessibility landscape of tumors in HCC. **a**. Different chromatin accessibility landscape between *C. sinensis*^+^ HCC tumors and *C. sinensis*^− ^HCC tumors. **b**. The top de novo motifs enriched in up and down accessibility peaks between *C. sinensis*^+^ HCC tumors and *C. sinensis*^− ^HCC tumors. **c**, **d**. Enrichment analysis of GO terms for up (**c**) and down (**d**) accessibility peaks between *C. sinensis*^+^ HCC tumors and *C. sinensis*^− ^HCC tumors. **e**. KEGG analysis of down accessibility peaks between *C. sinensis*^+^ HCC tumors and *C. sinensis*^− ^HCC tumors. **f**. IGV shows representative different accessibility peaks between *C. sinensis*^+^ HCC tumors and *C. sinensis*^− ^HCC tumors. *Cs* represents* C. sinensis*

To decipher the epigenetic mechanisms underlying the distinct responses of *C. sinensis* infection, the accessibility of regions in *C. sinensis*^+^ HCC tumors and *C. sinensis*^− ^HCC tumors were first compared. A total of 3599 increased- and 5797 decreased-accessibility regions were identified in *C. sinensis*^+^ HCC tumors compared with *C. sinensis*^*−*^ HCC tumors, accounting for 5.57% of the overall regions identified (Fig. 3a). The top 2 TFs whose motifs were enriched in increased-accessibility regions were HNF4A and FOXO1, and the top 2 TFs whose motifs were enriched in decreased-accessibility regions were ELF4 and RELA (Fig. 3b). GO enrichment analysis of DARs revealed that *C. sinensis*⁺ HCC tumors present increased chromatin accessibility at loci associated with cytoskeletal organization (e.g., actin binding) [38], polarized cellular architecture (apical part of the cell), and hormone responsiveness (response to peptide hormones) [39] (Fig. 3c). These features are indicative of enhanced cellular motility and structural plasticity, both of which are hallmarks of tumor aggressiveness and EMT. In contrast, regions with reduced accessibility were enriched for genes involved in immune-related biological processes such as lymphocyte differentiation, suggesting an epigenetically encoded immunosuppressive state (Fig. 3d). KEGG pathway analysis further supported this finding, revealing the downregulation of immune surveillance and checkpoint pathways (e.g., PD-L1, PD-1 signaling and T-cell receptor signaling), which is consistent with immune escape in *C. sinensis*⁺ tumors (Fig. 3e). Finally, two representative regions of DARs were exhibited (Fig. 3f).

**Fig. 4:**
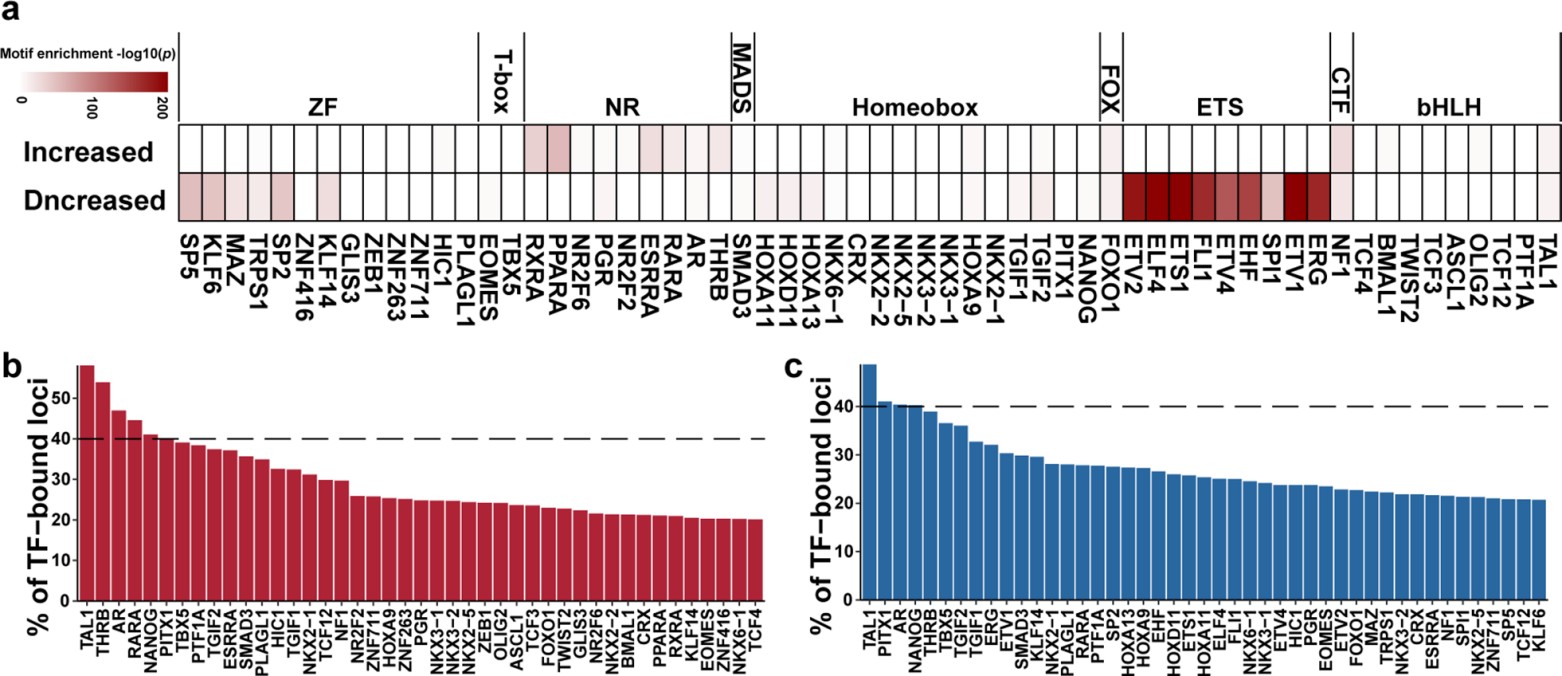
The potential core TFs in HCC after *C. sinensis* infection. **a**. TF motif enrichment in DARs. *P* values were calculated by HOMER. The color bar indicates Log10(p). **b**, **c**. Quantification of coverage of TF-binding motifs in increased (**b**) and decreased (**c**) DARs in *C. sinensis*^+^ HCC tumors compared with *C. sinensis*^−^ HCC tumors

To identify the key TFs responsible for modulating the dynamics of chromatin accessibility in response to *C. sinensis* infection, we analyzed the TF motifs associated with increased and decreased DARs in *C. sinensis*^+^ HCC tumors compared with those in *C. sinensis*^*−*^ HCC tumors. Increased DARs were enriched for NR TFs; however, decreased DARs were enriched mainly for ZF and ETS TFs (Fig. 4a). Moreover, five (TAL1, THRB, AR, RARA, and NANOG) and four (TAL1, PITX1, AR, and NANOG) TF motifs represented more than 40% of the increased and decreased DARs, TAL1, AR, and NANOG (Fig. 4b, c). TAL1, AR, and NANOG exhibited both upregulation and downregulation, suggesting that they may play dual roles in the dynamic regulation of chromatin accessibility induced by *C. sinensis* infection.

To explore whether *C. sinensis* infection also changed chromatin accessibility in tumor-adjacent tissues, relevant analyses were performed. A total of 1952 increased- and 1971 decreased-accessibility regions were identified in *C. sinensis*^+^ HCC tumor-adjacent tissues and *C. sinensis*^*− *^HCC tumor-adjacent tissues, accounting for 2.33% of the overall regions identified (Supplementary Fig. S3a). The top 2 TFs whose motifs were enriched in increased-accessibility regions were HNF4A and BORIS, and the top 2 TFs whose motifs were enriched in decreased-accessibility regions were SFI1 and FOSL1 (Supplementary Fig. S3b). Moreover, the GO analysis results revealed that increased-accessibility regions were enriched for genes involved in the apical part of the cell and the synaptic membrane, etc. (Supplementary Fig. S3c), and decreased-accessibility regions were enriched for genes involved in actin filament organization, the cell leading edge, and GTPase, etc. (Supplementary Fig. S3d). The KEGG pathways associated with regions with decreased accessibility showed enrichment only for cell adhesion molecules (Supplementary Fig. S3e).

### *C. sinensis* infection induces different expression profiles of tumors in HCC

We also obtained RNA-seq data from *C. sinensis*^+^ HCC and *C. sinensis*^−^ HCC tumors and identified the differentially expressed genes (DEGs). In total, 757 upregulated genes and 802 downregulated genes were found in *C. sinensis*^+^ HCC compared with *C. sinensis*^−^ HCC tumors (Supplementary Fig. S4a and Supplementary Table S3), indicating a dynamic expression profile between *C. sinensis*^+^ HCC and *C. sinensis*^−^ HCC tumors. GO analysis showed that the upregulated genes were enriched in inclusion body assembly, the collagen-containing extracellular matrix, and the extracellular matrix, etc. (Supplementary Fig. S4b). However, the downregulated genes were enriched in surface receptors, receptor complexes, and antigen binding, etc. (Supplementary Fig. S4c). In addition, KEGG analysis revealed that the downregulated genes were enriched in Th1 and Th2 cell differentiation and cell adhesion molecules, etc. (Supplementary Fig. S4d).

### Changes in chromatin accessibility correspond to the regulation of gene expression

**Fig. 5:**
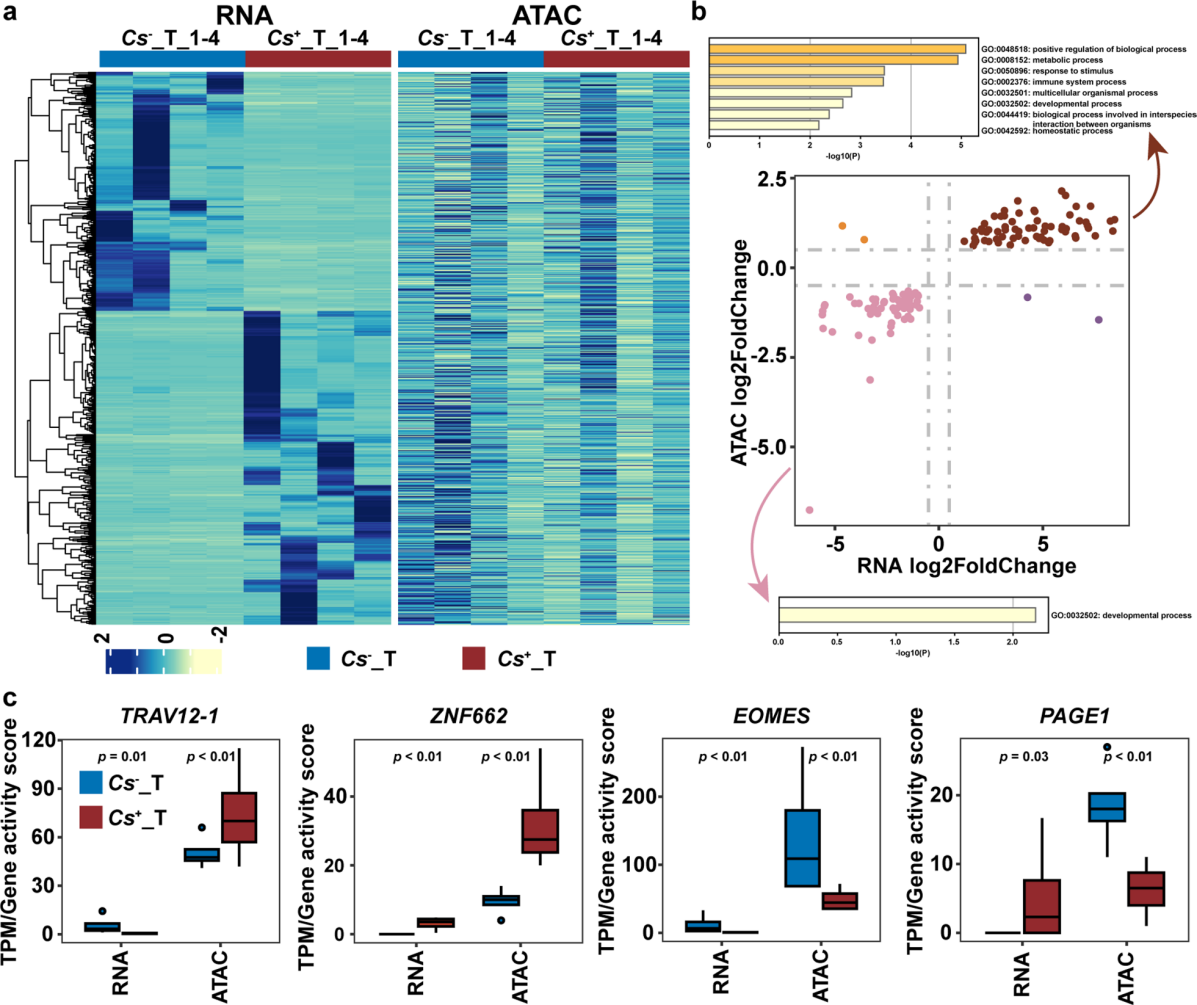
Joint analysis of RNA and ATAC from the same sample in the *C. sinensis*^+^ HCC tumors and *C. sinensis*^− ^HCC tumors. **a**. Heatmap representation of relative expression levels of genes that were differentially expressed between *C. sinensis*^+^ HCC tumors and *C. sinensis*^− ^HCC tumors. The relative changes of chromatin accessibility in the same sample along the same gene are shown to the right. **b**. Correlation of the RNA expression and promoter accessibility changes in the *C. sinensis*^+^ HCC tumors and *C. sinensis*^− ^HCC tumors. **c**. The RNA expression and promoter accessibility of four RNA expression and promoter accessibility genes. *Cs* represents* C. sinensis*

We next integrated ATAC-seq and RNA-seq data to investigate the relationship between chromatin accessibility and gene expression. Considering that dynamic changes in promoter accessibility directly affect the transcriptional activity of genes, differentially accessible promoter elements are of interest. The promoter regions were considered the regions within 3 kb upstream and downstream of the TSS, and a gene activity matrix was built (Supplementary Table S4). The heatmap illustrates the expression levels of all DEGs between *C. sinensis*^+^ HCC and *C. sinensis*^−^ HCC tumors (Fig. 5a). The gene activity scores of the corresponding genes are also presented. The trends of variation in the expression level and gene activity score of the same gene were not exactly the same.

To further explore the relationship between the expression levels of all DEGs and their corresponding promoter accessibility, we first computed the difference in gene activity scores between *C. sinensis*^+^ HCC and *C. sinensis*^−^ HCC tumors. The correlations between them are shown in Fig. 5b. In total, the expression levels of most genes were positively correlated with promoter accessibility. However, a few genes were negatively correlated. Finally, the expression levels and gene activity scores of four representative genes were determined (Fig. 5c).

### Variation in chromatin accessibility is correlated with histone modifications

**Fig. 6:**
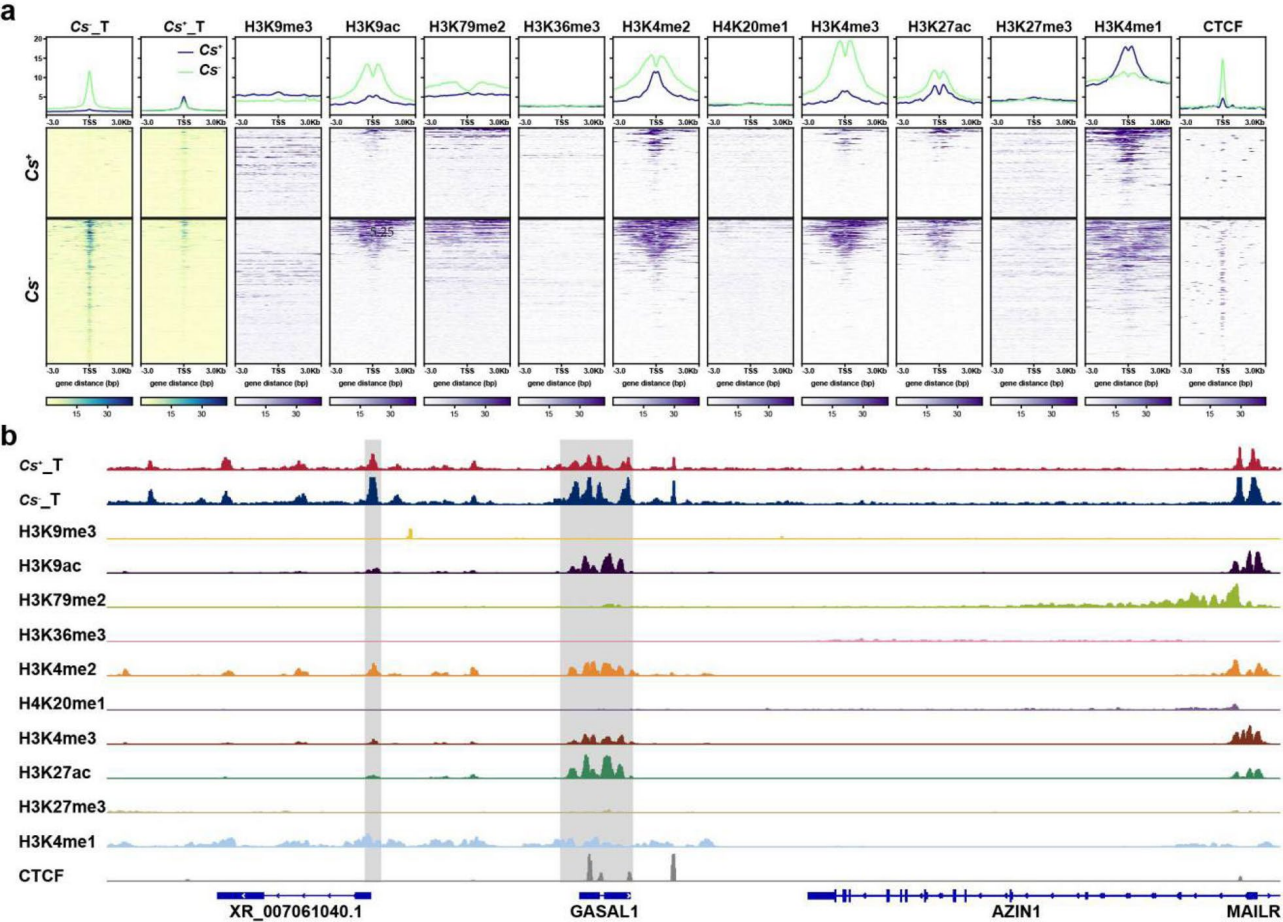
Joint analysis of ATAC-seq and public ChIP-seq data. **a**. Heatmap representation of the aggregated ATAC-seq and paired tag histone modification signals around different accessibility regions between *C. sinensis*^+^ HCC tumors and *C. sinensis*^− ^HCC tumors. **b.** IGV shows representative ATAC-seq and ChIP-seq signals. *Cs* represents* C. sinensis*

Many studies have shown a significant association between histone modifications and chromatin accessibility [40]. To explore which histone modifications are associated with changes in chromatin accessibility due to *C. sinensis* infection, the ChIP-seq data of the HepG2 cell line, including H3K9me3, H3K9ac, H3K79me2, H3K36me3, H3K4me2, H4K20me1, H3K4me3, H3K27ac, H3K27me3, H3K4me1, and CTCF, were downloaded from a public database. The enrichment signals of all ChIP-seq data in DARs were illustrated, and we found that H3K9ac, H3K4me2, H3K4me3, H3K27ac, and H3K4me1 were associated with chromatin accessibility (Fig. 6a). Notably, the signal for CTCF enrichment was also found in DARs, which suggested that *C. sinensis* infection would also change the three-dimensional structure of the HCC genome due to the relationship between CTCF and the spatial structure of chromatin. We also represented the ATAC-seq and ChIP-seq signals of representative regions (Fig. 6b and Supplementary Fig. S5).

### *C. sinensis* infection promotes the migratory behavior of HCC cells and is associated with increased metastatic potential

**Fig. 7:**
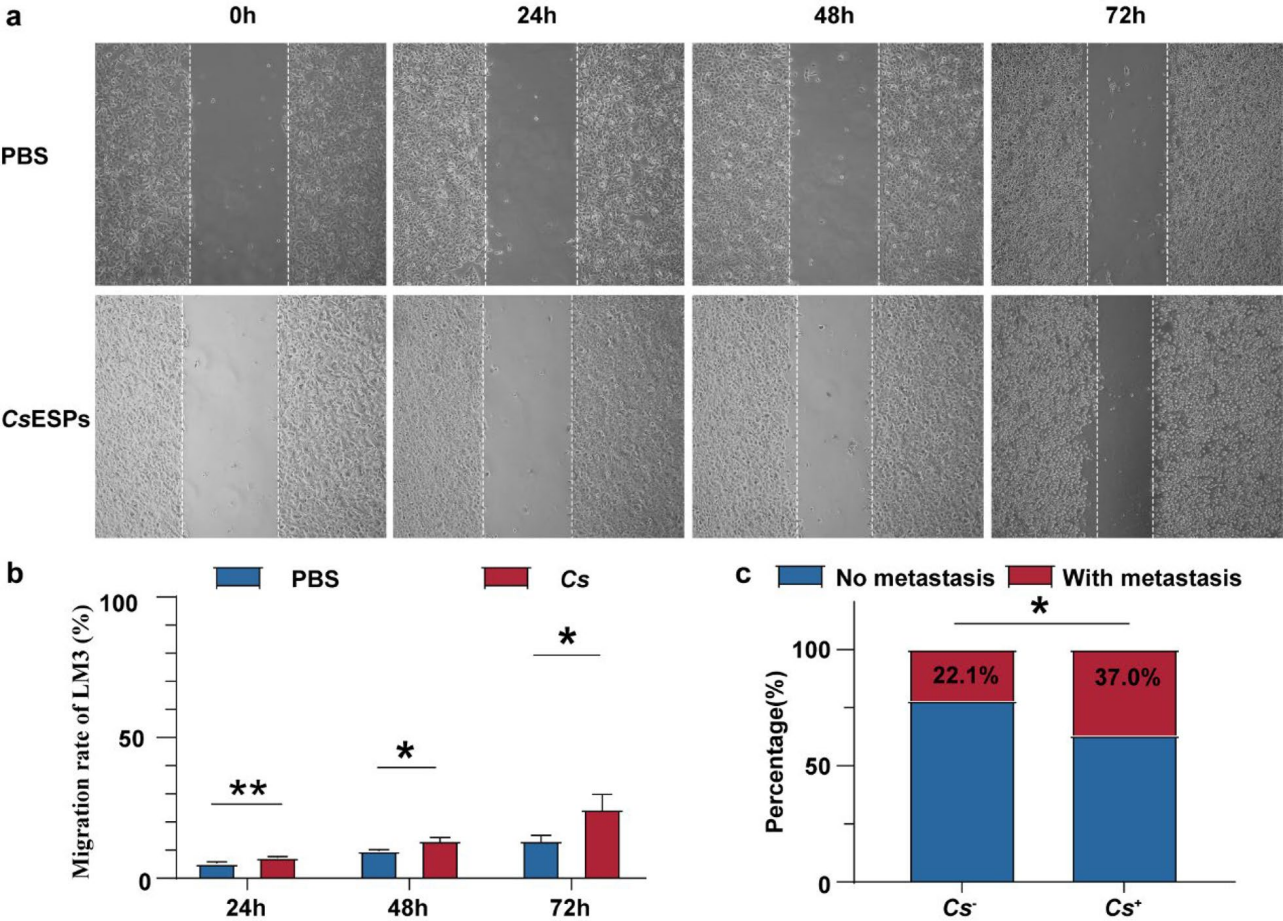
*C. sinensis* infection promotes the migratory behavior of HCC cells and is correlated with increased metastatic potential. **a** The scratch assay of HCCLM3 cells co-cultured with *Cs*ESPs or PBS. **b** Migration statistics of HCCLM3 cells co-cultured with *Cs*ESPs (N = 3) or PBS (N = 3). **c** Comparison of the metastasis percentage of *C. sinensis*^+^ (N = 92) and *C. sinensis*^−^ (N = 855) HCC patients. *P < 0.05, **P < 0.001. *Cs* represents* C. sinensis*

Our analysis revealed a correlation between *C. sinensis* infection and alterations in cell–substrate junctions (Fig. 3). Cell–substrate junctions engage with the extracellular matrix (ECM) through specific receptors, regulating cellular behavior and influencing cell migration and function [41–43]. Additionally, alterations in the ECM are closely associated with the invasion and metastasis of tumor cells [44], suggesting that *C. sinensis* infection may increase the aggressiveness of HCC. To test this hypothesis, we cocultured 50 μg/mL *Cs*ESPs with HCCLM3 cells. The results demonstrated that the migratory ability of HCCLM3 cells treated with *Cs*ESPs was significantly greater than that of those treated with PBS (Fig. 7a, b). Additionally, we conducted a retrospective analysis of clinical data from HCC patients. Statistical analysis revealed that the metastasis rate in *C. sinensis*^+^ HCC patients (N = 92) was significantly greater than that in *C. sinensis*^−^ HCC patients (N = 855), with rates of 22.1% and 37.0%, respectively (*P* = 0.02). These findings collectively support the notion that *C. sinensis* infection promotes HCC cell migration and is associated with increased metastatic potential in HCC.

## Discussion

Previous studies have demonstrated that *C. sinensis* is related to poor prognosis in patients with HCC, but the specific mechanisms and mediators involved are not fully understood [8–11, 45]. In this study, we integrated multiple sequencing techniques, including ATAC-seq, RNA-seq, and ChIP-seq, to comprehensively investigate changes in chromatin accessibility, alterations in gene expression, and their associations with histone modifications and clinical phenotypes. These findings may substantially advance our understanding of the pathogenesis of *C. sinensis*-associated hepatocellular carcinoma and offer new perspectives for the identification of diagnostic biomarkers and the development of targeted therapeutic strategies.

In our study, one of the most significant findings was that *C. sinensis* infection remodels the chromatin landscape in HCC. Chromatin accessibility reflects a complex interplay of regulatory elements involving enhancers, promoters, insulators, and chromatin-binding factors, all of which synergistically regulate gene expression [46]. Chromatin regions with increased accessibility in *C. sinensis*⁺ tumors were significantly enriched for the transcription factor motifs of HNF4A and FOXO1. HNF4A is the principal regulatory factor in the liver and governs several essential metabolic pathways, including glycolysis, gluconeogenesis, fatty acid oxidation, and bile acid production [47]. However, FOXO1 functions downstream of insulin signaling to mediate glucose metabolism and gluconeogenesis [48]. GO analysis further revealed significant enrichment of peptide hormone response pathways, which have been implicated in DNA damage repair and metabolic reprogramming in cancer [39, 49, 50]. These epigenetic findings align with recent metabolomic data. Animal studies have demonstrated that *C. sinensis* infection alters hepatic metabolism, particularly fatty acid oxidation, amino acid metabolism, and bile acid synthesis [51, 52]. Tang et al. reported that serum from HCC patients infected with *C. sinensis* presented a reduction in essential metabolites, including argininosuccinate synthase and glucose, alongside global metabolic reprogramming [53]. Tingzheng Zhan et al. demonstrated significant alterations in metabolism-related pathways in a murine model of *C. sinensis* infection, which were closely associated with the progression of liver fibrosis and cirrhosis [54]. Similarly, Xu et al. reported that, in *C. sinensis*-associated intrahepatic cholangiocarcinoma (iCCA), *C. sinensis* altered the tumor metabolic microenvironment by stimulating fatty acid synthase (FASN)-mediated fatty acid production [55]. Our epigenetic findings align closely with the latest metabolomic discoveries, offering chromatin-level evidence for a possible regulatory foundation enabling metabolic reprogramming. However, further in-depth investigations are warranted to validate and fully elucidate these potential regulatory relationships.

Interestingly, beyond metabolic disruption, regions of decreased chromatin accessibility in *C. sinensis*⁺ samples were enriched for the RELA and ELF4 TFs. ELF4, a member of the ETS family of TFs, has been shown to facilitate the production of type I interferons [56]. Previous studies have demonstrated that Elf4-deficient mice exhibit significant impairments in the development and function of natural killer (NK) cells and NK-T cells, highlighting the critical role of Elf4 in the immune system [57]. RELA, a key member of the NF-kB family, commonly forms a heterodimer with p50 to regulate the expression of genes involved in inflammation, immune responses, cell proliferation, and survival [58]. Notably, recent studies have proposed that, after severe or chronic infections, hosts may experience a long-term phenomenon of “epigenetic suppression,” leading to persistently low immune function [59]. Our enrichment analysis revealed that regions with reduced chromatin accessibility were significantly associated with immune-related GO terms such as lymphocyte differentiation. KEGG pathway analysis further revealed significant enrichment of key immune regulatory pathways, including the PD-1/PD-L1 signaling pathway and the T-cell receptor signaling pathway. These findings suggest that *C. sinensis* infection may suppress antitumor immunity via epigenetic mechanisms. Consistent with these findings, studies have confirmed that *C. sinensis* infection significantly reduces the expression of Th1/Th2/Th17 cytokines such as TNF-α, IL-1β, and IFN-γ in the host serum, resulting in an overall immunosuppressive state [60]. Zhao et al. reported that adult *C. sinensis* worm proteins promote a Th2-skewed immune response by modulating dendritic cell function [61]. Similarly, clinical multiomic analyses by Tang et al. revealed that *C. sinensis* infection induces inflammatory responses while facilitating tumor immune escape [53]. Importantly, Xu et al. further demonstrated that increased lipid metabolism in *C. sinensis*-associated iCCA contributes to an immunosuppressive tumor microenvironment and that this effect can be reversed by FASN inhibition to increase the efficacy of anti–PD-1 immunotherapy [55]. In summary, our data suggest that decreased chromatin accessibility at RELA/ELF4-binding sites may contribute to long-term immune suppression in *C. sinensis*-infected hosts. While the specific functional consequences remain to be fully elucidated, these findings raise the possibility that epigenetic mechanisms may play a role in parasite-associated immune regulation, offering a new perspective for further investigations into *C. sinensis*–induced immune modulation.

*C. sinensis* infection appears to orchestrate extensive remodeling of tumor cell architecture and transcriptional regulation. ATAC-seq analysis revealed that chromatin regions with increased accessibility in *C. sinensis*^+^ tumors were significantly enriched for genes involved in actin binding, indicating enhanced cytoskeletal dynamics and cellular motility—hallmarks of EMT [62]. Notably, FOXO1, an enriched transcription factor in these regions, is known to regulate oxidative stress responses, cell cycle arrest, and EMT using the PI3K/AKT pathway [63]. Consistent with our findings, previous studies have linked FOXO1 overexpression to more aggressive phenotypes in HCC [64, 65]. Transcriptomic profiling further corroborated these epigenetic signatures. The genes upregulated in *C. sinensis*^+^ tumors were enriched in pathways related to inclusion body formation, collagen-containing extracellular matrix organization, and tissue structural remodeling, which are closely associated with fibrosis and aggressive tumor phenotypes. Conversely, the downregulated genes were predominantly involved in immune-related processes such as antigen binding, receptor signaling, and immune receptor complex formation, suggesting a shift toward an immunosuppressive transcriptional program. In addition to changes in accessibility and transcription, *C. sinensis* infection also appears to influence higher-order chromatin architecture. DARs were enriched for H3K27ac, an active enhancer marker [66], indicating that *C. sinensis* may reshape enhancer landscapes in HCC cells. Furthermore, enrichment of CTCF-binding sites within these DARs points to potential perturbations in topologically associating domain (TAD) boundaries. Since CTCF and the cohesin complex maintain TAD integrity and transcriptional insulation [67], alterations in CTCF occupancy may drive TAD fusion or reorganization, thereby disrupting gene regulatory networks. Collectively, these findings demonstrate that *C. sinensis* infection has multiple regulatory effects, including alterations in cytoskeletal dynamics, immune evasion, enhancer activity, and 3D genome organization, thus advancing our understanding of its epigenetic role in HCC progression.

Overall, our results indicate that *C. sinensis* is associated with altered transcription factor binding at loci involved in metabolic reprogramming, immune modulation, and cytoskeletal dynamics, which may contribute to tumor progression. However, owing to the limited sample size and the lack of direct functional validation and chromatin interaction data, these associations should be interpreted with caution. Future studies should incorporate larger cohorts alongside CRISPR-based functional perturbations, transcription factor knockdown, and protein-level assays to determine causality and uncover the precise mechanisms underlying these epigenetic alterations.

## Conclusions

Our study reveals that *C. sinensis* infection is associated with extensive chromatin remodeling in HCC, affecting key pathways related to metabolism, immune regulation, and cytoskeletal dynamics. These epigenetic alterations may contribute to tumor progression by promoting metabolic reprogramming, immune suppression, and invasive potential. While further validation is needed, our findings provide novel mechanistic insights into parasite-associated HCC and highlight potential targets for future diagnostic and therapeutic strategies.

## Supplementary Information

Additional file 1 (Quality assessment of ATAC-seq data.)

Additional file 2 (Chromatin accessibility landscape of *C. sinensis*^–^HCC.)

Additional file 3 (*C. sinensis* change the chromatin accessibility landscape of tumor-adjacent tissues in HCC.)

Additional file 4 (*C. sinensis* change the expression profiles of tumors in HCC.)

Additional file 5 (IGV shows representative ATAC-seq and ChIP-seq signals.)

Additional file 6 (XLSX 22842 KB)

## Data Availability

The raw sequencing data have been deposited in GEO under accession number GSE276855 and in GSA-Human under accession number HRA009797 (subHRA014148).The ChIP-seq data was downloaded from GEO (GSE29611).

## Abbreviations

HCC: Hepatocellular carcinoma
iCCA: Intrahepatic cholangiocarcinoma
IARC: International Agency for Research on Cancer
*C. sinensis*: *Clonorchis sinensis*
*Cs*GRN: *C. sinensis* Granulin
*Cs*ESPs: *C. sinensis* Excretory/secretory products
*C. sinensis*^+^: *C. sinensis*-infected
*C. sinensis*^−^: Non-*C. sinensis*-infected
ATAC-seq: Assay for transposase-accessible chromatin with high-throughput sequencing
RNA-seq: RNA sequencing
SD: Sprague–Dawley
UNG: Uracil-N-glyosylase
*C. sinensis*^+^_T: *C. sinensis*^+^ HCC tumor
*C. sinensis*^+^_P: *C. sinensis*^+^ Adjacent tissue
*C. sinensis*^−^_T: *C. sinensis*^−^ HCC tumor
*C. sinensis*^−^_P: *C. sinensis*^−^ Adjacent tissue
DEGs: Different expression genes
ECM: Extracellular matrix
FASN: Fatty acid synthase
