# Supplementary figures and images for: Clonorchis sinensis infection remodels chromatin accessibility in hepatocellular carcinoma
Source: Parasit Vectors. 2025 Jul 10;18:276. doi: 10.1186/s13071-025-06909-6 (PMC12247385; doi:10.1186/s13071-025-06909-6)

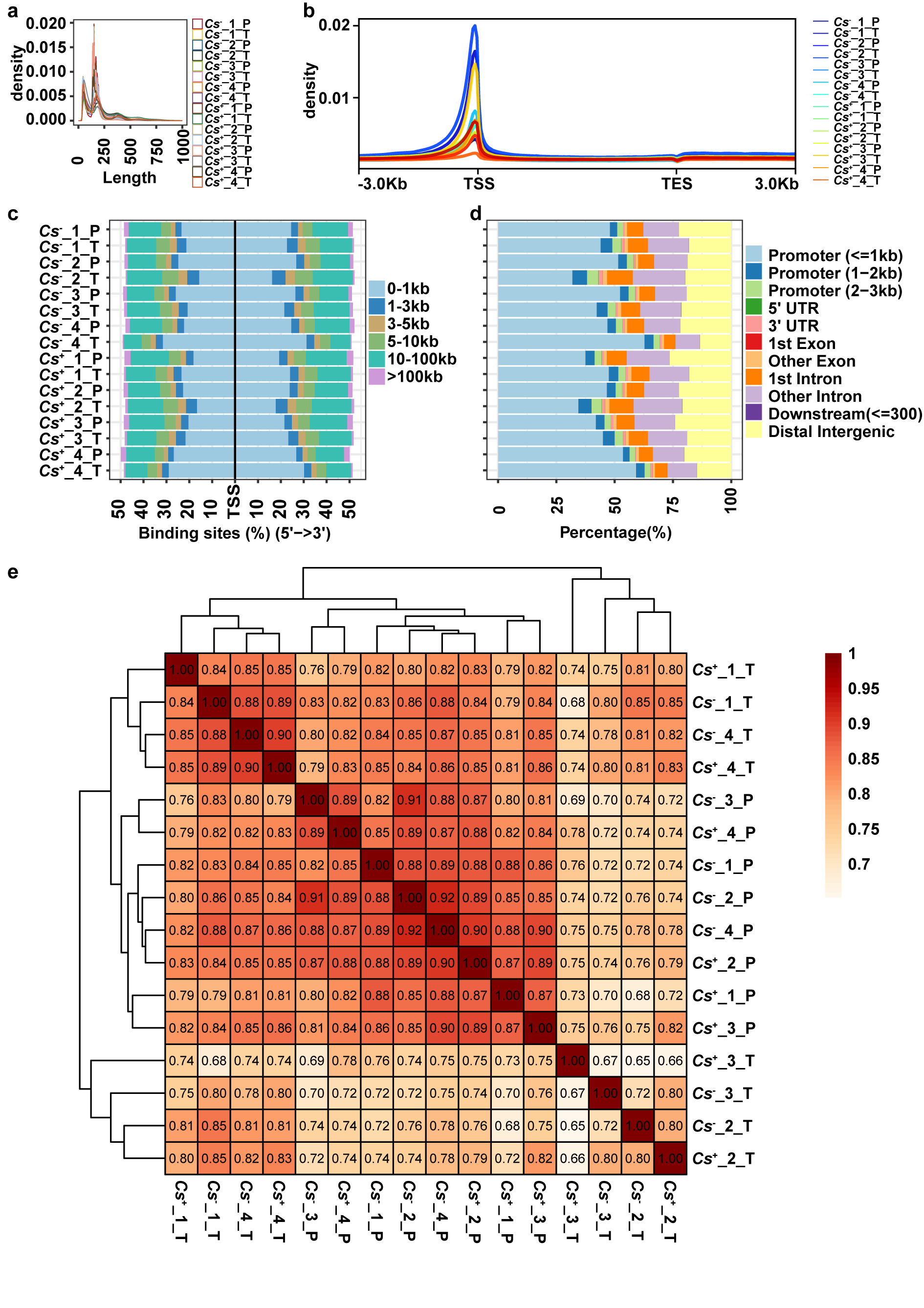

Supplement: Supplementary file 1 — Additional file 1 (Quality assessment of ATAC-seq data.) [file 13071_2025_6909_MOESM1_ESM.tif]

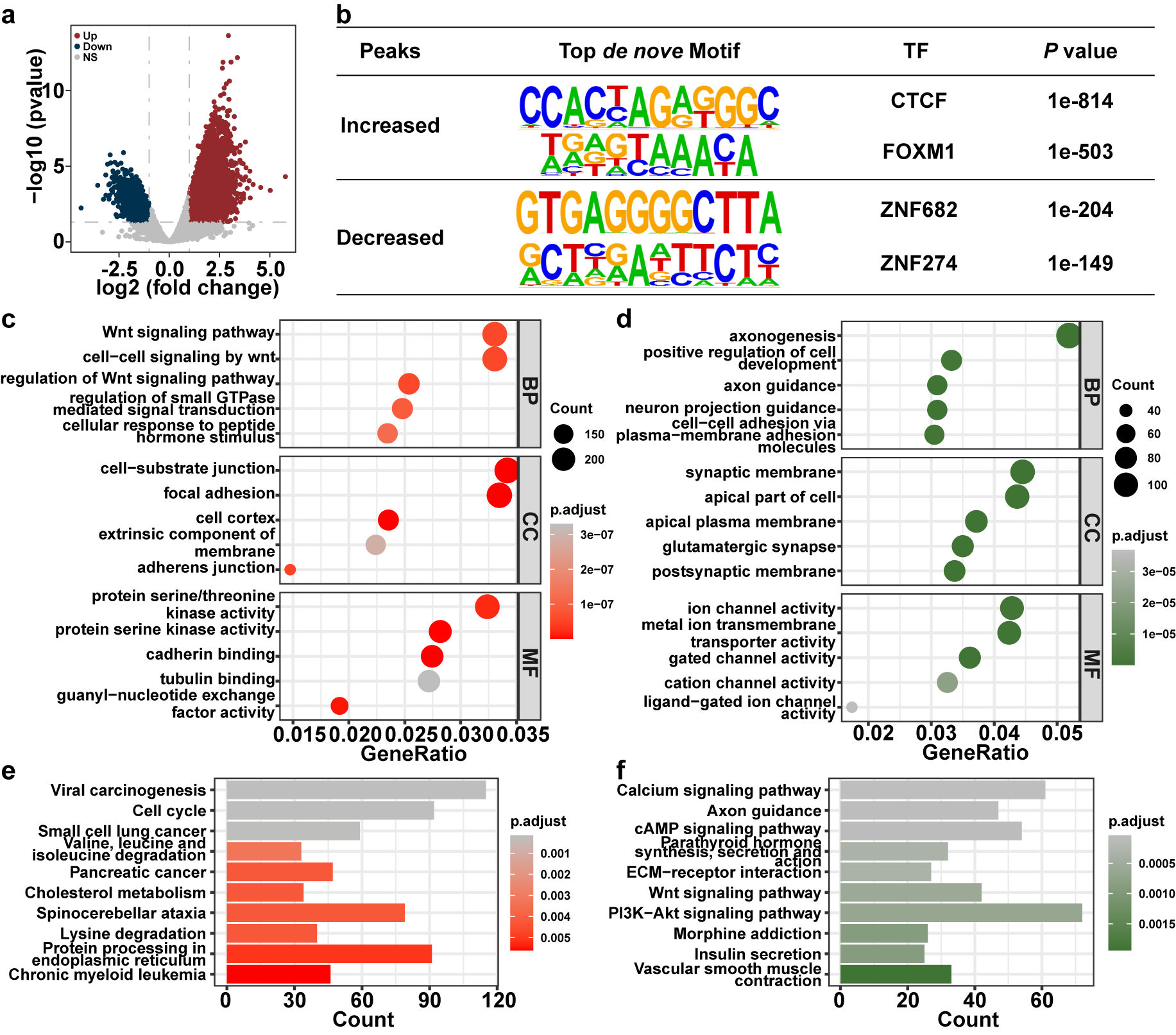

Supplement: Supplementary file 2 — Additional file 2 (Chromatin accessibility landscape of C. sinensis–HCC.) [file 13071_2025_6909_MOESM2_ESM.tif]

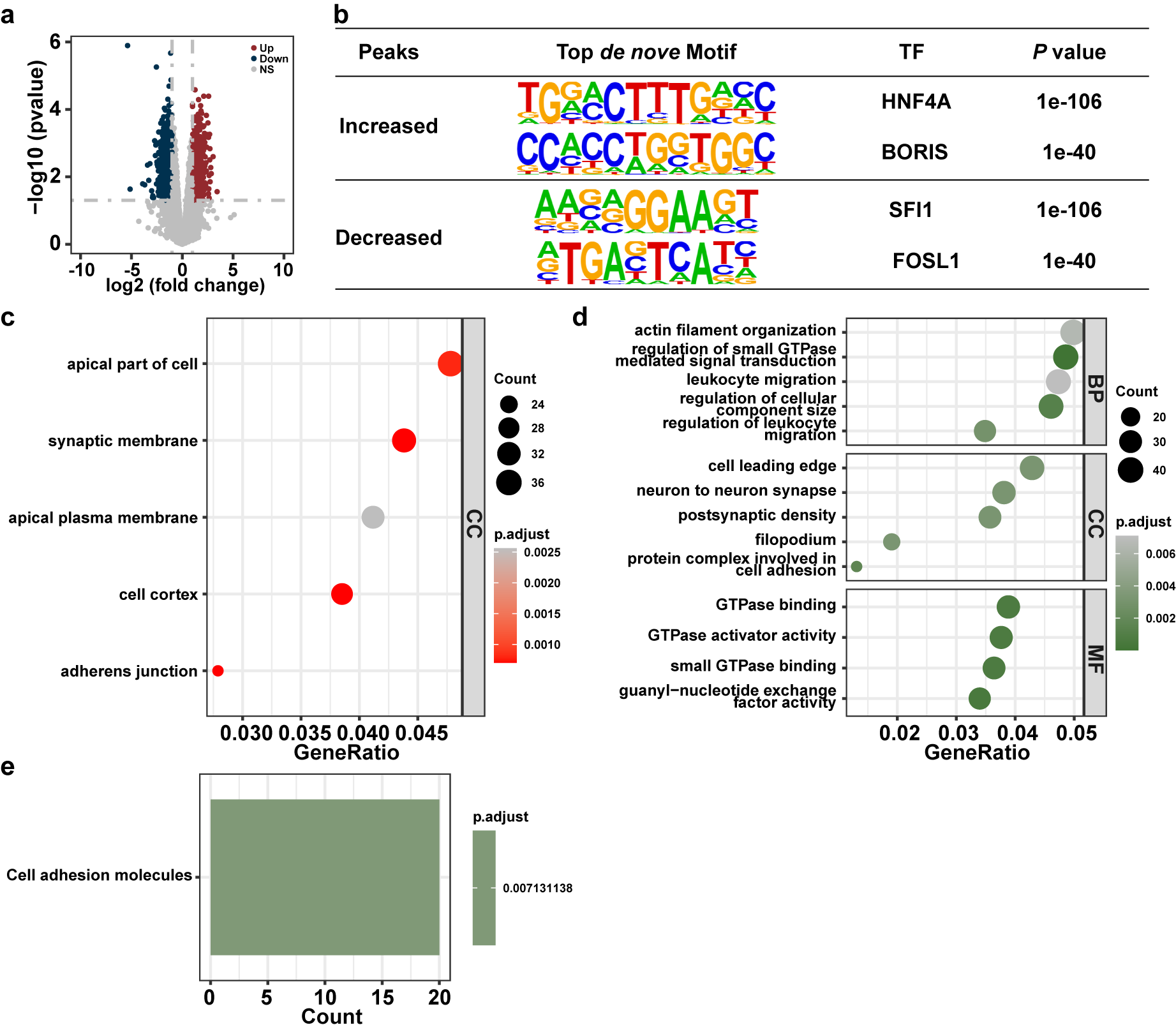

Supplement: Supplementary file 3 — Additional file 3 (C. sinensis change the chromatin accessibility landscape of tumor-adjacent tissues in HCC.) [file 13071_2025_6909_MOESM3_ESM.tif]

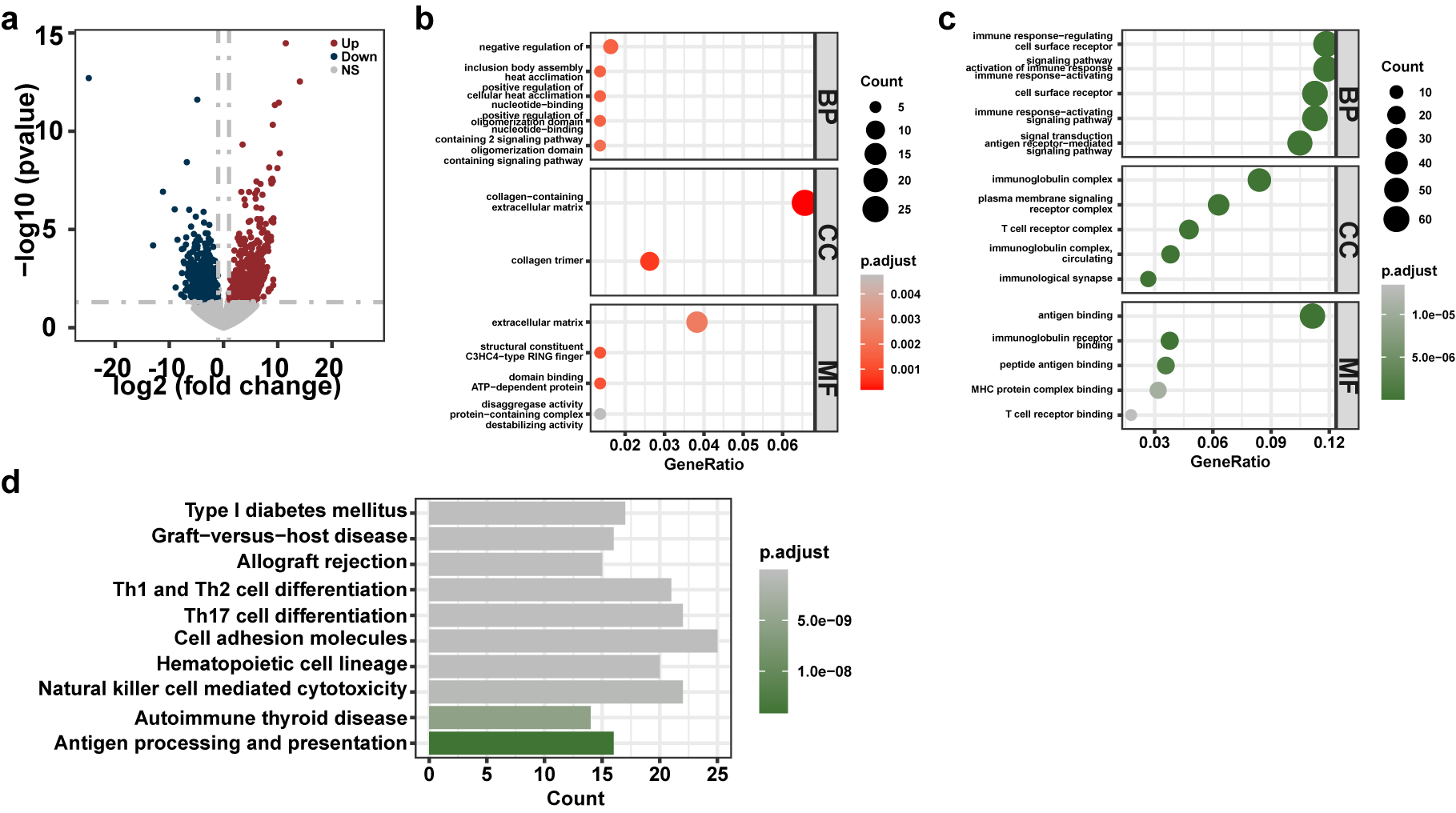

Supplement: Supplementary file 4 — Additional file 4 (C. sinensis change the expression profiles of tumors in HCC.) [file 13071_2025_6909_MOESM4_ESM.tif]

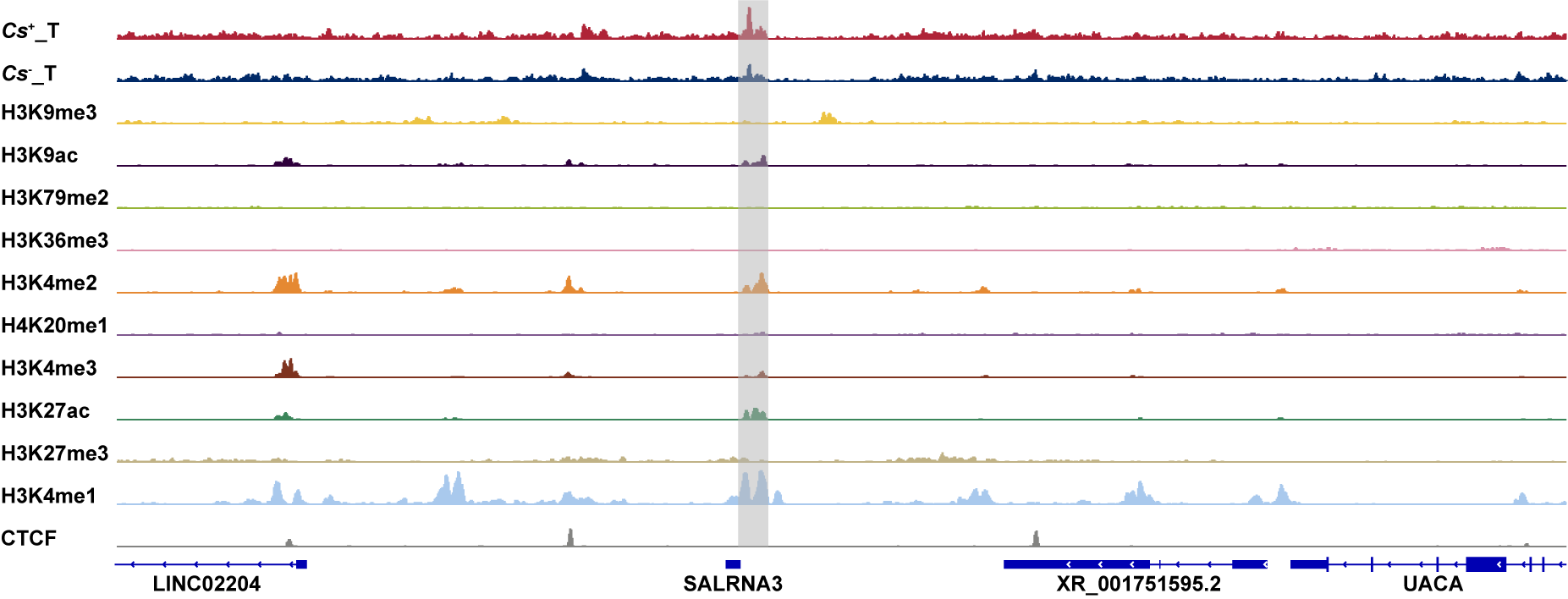

Supplement: Supplementary file 5 — Additional file 5 (IGV shows representative ATAC-seq and ChIP-seq signals.) [file 13071_2025_6909_MOESM5_ESM.tif]
